# Knowledge, attitudes, practices, and beliefs regarding prenatal alcohol consumption among women in Leyte, the Philippines

**DOI:** 10.3389/fgwh.2023.1156681

**Published:** 2023-08-09

**Authors:** Alice M. Huang, Matthew N. Neale, Spencer C. Darveau, Marianne J. Sagliba, Amabelle J. Moreno, Maria Paz G. Urbina, Veronica Tallo, Emily A. McDonald, Mario A. Jiz, Jennifer F. Friedman

**Affiliations:** ^1^ The Warren Alpert Medical School of Brown University, Providence, RI, United States; ^2^ Research Institute for Tropical Medicine, Manila, Philippines; ^3^ Center for International Health Research at Rhode Island Hospital, Providence, RI, United States; ^4^ Department of Pediatrics, The Warren Alpert Medical School of Brown University, Providence, RI, United States

**Keywords:** alcohol, prenatal, LMIC, FASD, tuba, KAPB

## Abstract

**Objectives:**

Fetal alcohol spectrum disorder (FASD) captures the broad range of emotional, cognitive, behavioral, and congenital abnormalities associated with maternal alcohol consumption, and women living in resource-limited settings may be higher risk. This study aims to examine knowledge, attitudes, practices, and beliefs (KAPB) of women in Leyte, The Philippines regarding prenatal alcohol consumption.

**Methods:**

One hundred postpartum women were recruited from a birth cohort in Leyte. A prenatal alcohol use KAPB survey was constructed in Waray, the local language. The survey was administered in June-September 2019. Descriptive statistics, chi-squared test, and Fisher's exact test were used to analyze responses.

**Results:**

Seventy-five percent of subjects reported drinking tuba, a local palm wine, during pregnancy. Most participants (75%) did not believe tuba contained alcohol. Women who believed tuba contains no alcohol were more likely to drink tuba during pregnancy (81.3%) than women who believed tuba contains alcohol (56.0%), *X*^2^(1, *N* = 100) = 6.41, *p *= .011. Women who drank tuba during pregnancy were more likely to believe tuba has health benefits (60%) than women who did not drink tuba during pregnancy (12%), Fisher's exact *p* < .05, citing increased red blood cell count and unproven antiparasitic qualities. Fifteen percent of subjects reported having fed their babies tuba. Nearly all (98%) were willing to attenuate tuba/alcohol consumption if told that this practice negatively impacts pregnancies.

**Conclusion:**

Misinformation about tuba appears widespread in Leyte. Educating women of reproductive age in Leyte regarding prenatal tuba use may lead to a reduction in tuba use.

## Detailed key findings/implications

• Currently, little is known about prenatal alcohol consumption and FASD in low- and middle-income countries (LMICs). FASD is entirely preventable if pregnant women abstain from alcohol during pregnancy. It is likely that women in resource limited settings, such as Leyte, The Philippines, may be at high risk of giving birth to children with FASD due to a lack of systemic preventative education surrounding alcohol use in pregnancy, later confirmation of pregnancy, and consumption of unregulated alcohol brews that are locally made. This study examines the knowledge, attitudes, practices, and beliefs surrounding prenatal alcohol consumption, with a focus on tuba, a local, unregulated and commonly consumed palm wine in Leyte, The Philippines.
• Our findings are potentially generalizable to other regions of the world, specifically LMICs, where similar practices exist. Per this study, many women may be unaware that locally fermented beverages contain alcohol and that alcohol may harm the fetus. Thus, opportunities exist to target interventions that address this knowledge gap, helping decrease the risk of fetal alcohol spectrum disorder worldwide.
• Misinformation about tuba as an alcoholic beverage appears to play a role in the practices surrounding maternal and pediatric tuba consumption in Leyte, The Philippines. Most participants in our study reported consumption of alcohol during pregnancy. Future work should focus on incorporating tuba screening into already existing structures for alcohol and tobacco smoking screening at prenatal visits. Data from this study can inform local health departments in the creation of health education materials and/or programs addressing prenatal alcohol use for women of childbearing age.

## Introduction

In 1973, “fetal alcohol syndrome” (FAS) was coined to describe the cluster of birth defects with lifetime consequences due to prenatal alcohol exposure, including craniofacial abnormalities, growth restriction, and intellectual disabilities (1–3). Since then, the umbrella term “fetal alcohol spectrum disorder” (FASD) has been adopted to capture the broad range of emotional, cognitive, behavioral, and congenital abnormalities associated with prenatal alcohol exposure (1, 3–6). A number of large meta-analyses have estimated the global prevalence of FASD; among children and youth in the general population this has been estimated to be 7.7 per 1,000 (7) and among the general population, 1.46 per 1,000 (8). Of relevance to this study, some of the highest rates of FASD globally have been noted in LMIC nations (65.2–74.2 per 1,000 children) (9). Notably, this disorder is entirely preventable if pregnant women abstain from alcohol consumption (6).

Mothers of children with FASD are often shamed for having what is viewed as problematic patterns of alcohol use (10). The belief that pregnant women drink alcohol despite knowing its effects on their fetuses ignores complex sociocultural factors at play. Population and qualitative studies have shown that many mothers of children with FASD do not have alcohol use disorder (11–13). For example, a qualitative study conducted in New Zealand involving biological mothers living with their FASD-affected children revealed that many were unaware of the effects of prenatal alcohol exposure and had no knowledge of the potential risk of alcohol intake while pregnant (12). Systematic reviews across many settings reveal multiple reasons for pre-natal alcohol consumption including lack of awareness of harm and even perceived benefit, peer and cultural influences that continue during pregnancy, and others (14, 15). Additional risk factors for giving birth to children with FASD include lower maternal education level, lower socioeconomic status, paternal drinking and drug use at the time of pregnancy, reduced access to antenatal care and services, inadequate nutrition, and a poor developmental environment (e.g., stress, abuse, neglect), among other factors (16).

The global prevalence in the general population of consuming any quantity of alcohol during pregnancy has been estimated to be 9.8% (8). It is likely that women living in resource-limited settings, specifically in LMICs, may be at higher risk of giving birth to children with FASD. This increased risk is multifactorial and includes factors such as lack of systematic preventative education surrounding alcohol use during pregnancy and potential harms, later confirmation of pregnancy, and consumption of unregulated alcohol in the form of local or homemade alcoholic brews produced and sold outside of government control and without warning labels (17–19). This is pertinent to Leyte, The Philippines, where the most commonly consumed alcohol is a largely unregulated alcoholic beverage—a locally fermented palm wine called tuba (20, 21). We previously analyzed two tuba samples from Leyte to quantify the alcohol content: one from a home in one of our study villages and the other from a local shop. Both samples contained 7.3% ethanol by gas chromatography.

In an NIH funded, randomized-controlled trial in Leyte, The Philippines, over 75% of subjects reported continued alcohol consumption at 12–16 weeks gestation (20). No studies to our knowledge have examined this population to determine what underlying factors result in such high alcohol consumption rates. This study employed a survey assessing the knowledge, attitudes, practices, and beliefs (KAPB) surrounding prenatal alcohol consumption in Leyte, The Philippines in order to better understand what factors may influence mothers' decisions to consume alcohol during pregnancy.

## Methods

### Study population

Participants were recruited from an ongoing NIH-funded longitudinal birth cohort designed to examine the interactions among alcohol, helminth infections, and undernutrition in mediating adverse pregnancy outcomes (NIH R01AA024092). The main study enrolled 400 expectant mothers from over 50 villages served by 8 municipal health centers in northeastern Leyte, The Philippines. Participants were deemed eligible if they were otherwise healthy, ≥18 years of age, and had a verified singleton intrauterine pregnancy. Eligibility criteria were determined by history, physical exam, ultrasound, and laboratory assessment. For this study, a convenience sample of *N *= 100 women from the main study were sequentially enrolled during their child's planned follow-up visits (6–24 months postpartum) from June to September 2019 when staff were available to administer the questionnaire during these scheduled visits. All subjects provided their informed consent prior to inclusion.

### Knowledge, attitudes, practices, and beliefs (KAPB) survey

The survey was administered during the child's follow-up visits at the Research Institute of Tropical Medicine's satellite laboratory in Palo, Leyte, The Philippines from June to September 2019. (See appendix for full survey.) The survey was constructed using the instrument “Alcohol and Pregnancy Questionnaire” as a scaffold under express permission from the author (22). The KAPB survey was developed and greatly modified with input from local stakeholders in order to increase relevance to the study population and account for participant literacy levels. Initially constructed in English, the survey was translated into written form in Waray, the native language of the Eastern Visayas. Translation was jointly performed by multiple native speakers of Waray and back-translated to ensure accuracy. The questionnaire was then verbally administered by trained staff due to variability in literacy rates among participants. The questionnaire was initially pilot tested with six mothers and was subsequently modified to ensure clarity and survey comprehension. The instrument was comprised of 28 close- and open-ended questions. Closed ended questions were included (a) true/false, (b) yes/no, (c) rating a scale from 1 to 4 from “not important at all” to “very important, “ and d) selected choices such as type of alcoholic beverages which always included an “other” option. The following domains and the number of questions asked for that domain included: (1) perceived importance of maternal health during pregnancy as a means of promoting fetal health, (*N* = 6) (2) knowledge that tuba is an alcoholic beverage (*N* = 1), (3) self-reported behaviors regarding alcohol consumption before and during pregnancy (*N* = 6), (4) perceived risks or benefits of alcohol consumption during pregnancy (*N* = 4), (5) behaviors regarding infant alcohol consumption (*N* = 3), (6) perceived risks or benefits of infant alcohol consumption (*N* = 2), (7) receptiveness towards behavior change if informed of the negative effects of alcohol (*N* = 2), (8) potential influence from external sources (family, friends, and physicians) regarding tuba consumption (*N* = 2), and (9) perceived interest in gaining additional information regarding maternal and fetal health (*N* = 2). For open-ended questions, responses were aggregated into categories based on common themes. Of note, question 13 was limited to 90 respondents due to delayed addition of the question to the instrument.

### Statistical analysis

Survey data was recorded and managed using Microsoft Excel and Filemaker Pro (Claris, Santa Clara, CA). Descriptive statistics along with Pearson's *χ*^2^ tests were utilized to analyze survey response data. For proportions with smaller numbers in a specific subgroup or “cell” Fisher's Exact testing was employed. For all analyses, a *p*-value of <.05 was considered significant. Stata statistical software version 16 (StataCorp LP, College Station, TX) was used for data management and statistical analyses.

### Ethical statement

The study was approved by the Institutional Review Board at Rhode Island Hospital and The Ethical Review Board of the Research Institute of Tropical Medicine in Manila, The Philippines.

## Results

One hundred women participated in the study. Participants resided in one of 39 barangays, or villages, in three different municipalities in the province of Leyte, The Philippines: Alang-Alang, Jaro, and Santa Fe. Table 1 lists the sociodemographic characteristics of participants as captured during their pregnancy during the main study.

**Table 1:** Sociodemographic characteristics of the participant (*N* = 100).

Variables	*N*	%
Mother's age at time of survey (years)		
Mean	28.6 (5.3)	
Minimum, Maximum	21, 44	
Median	28	
Highest education^a^		
None	1	1
Elementary	25	25
High school	61	61
College	11	11
Vocational School	2	2
Does participant smoke?^a^		
Yes	0	0
No	100	100
Did participant smoke in the past?^a^		
Yes	8	8
No	92	92
Does participant drink tuba/alcohol?^a^		
Yes	69	69
No	31	31
If yes, how many years drinking?^a^		
Less than a year	7	7
1–5	46	46
6–10	12	12
11–15	1	1
16–20	3	3

^a^ Signifies data captured prenatally (i.e., upon initial enrolment of subjects in longitudinal birth cohort study).

**Figure 1:**
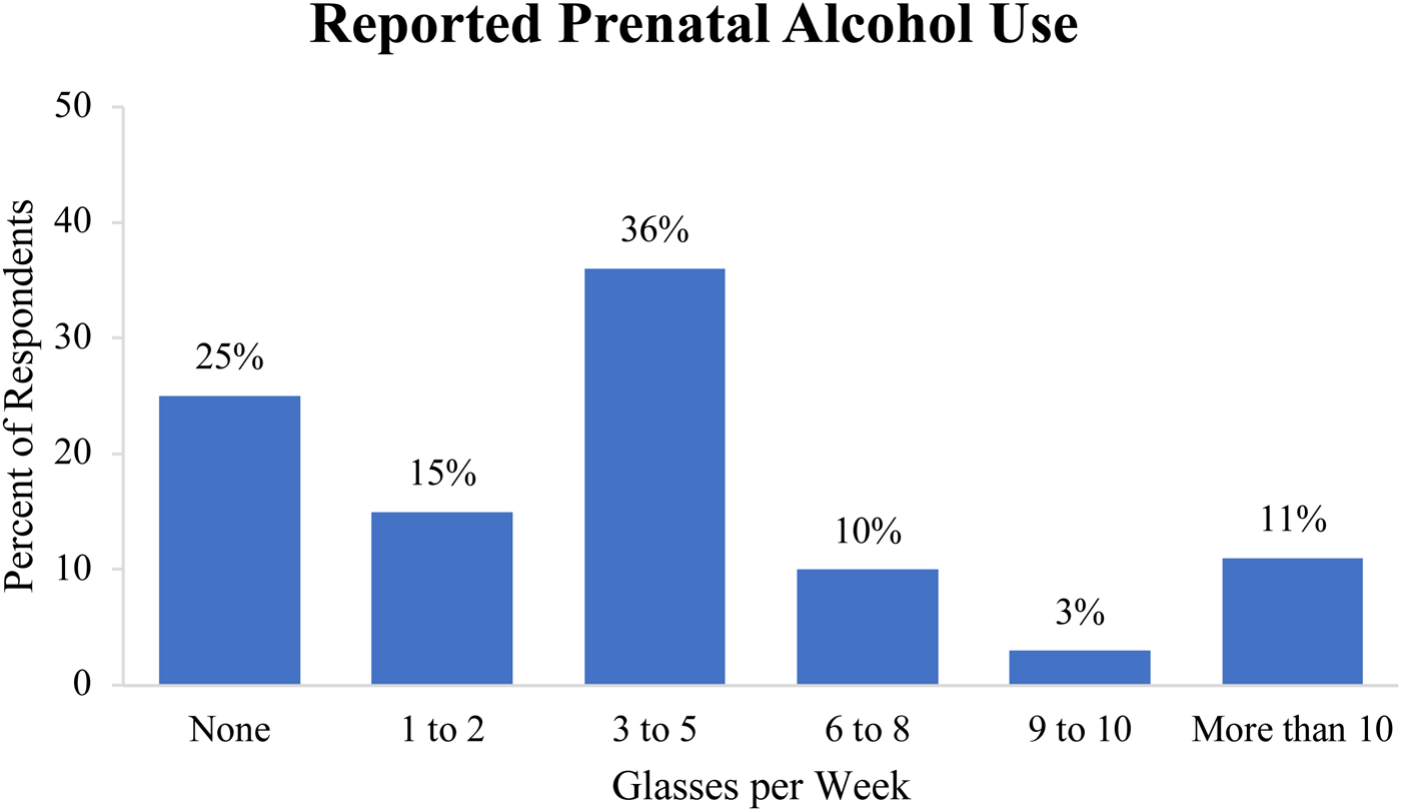
Self-reported estimates of weekly prenatal tuba or other alcohol consumption in glasses per week.

During our survey, 75 women (75%) reported drinking tuba or other alcoholic beverages during pregnancy. Of the women who reported drinking tuba or other alcoholic beverages, 100% reported specifically drinking tuba during pregnancy while only 5.3% (*n *= 4) reported drinking beer. Participants did not endorse consuming any other form of alcohol during pregnancy. Self-reported estimates of weekly prenatal tuba or other alcohol consumption are noted in Figure 1.

Participants were surveyed regarding the perceived importance of reducing alcohol consumption relative to other known healthy behaviors (Table 2). Almost all (97%) of mothers considered nutrition, exercise/physical activity, visiting a doctor or health professional, and reducing/stopping smoking to be either “important” or “very important,” while only 60% of participants considered decreasing the consumption of tuba or other alcoholic beverages during pregnancy “important” or “very important.”

**Table 2:** Perceived importance of various health-related behaviors during pregnancy.

Health Pregnancy Actions	Very important	Important	Not very important	Not important at all
*“How important was it for you to be healthy during your pregnancy to make it more likely for your baby to be born healthy?”*	66	34	0	0
*“I’m going to read some things that pregnant women might or might not do to make it more likely that their baby is born healthy. Tell me if you feel that these things are very important, important, not very important, or not important at all.”*				
Visit a doctor or health professional	77	22	1	0
Eat well/have good nutrition	80	20	0	0
Exercise/perform physical activity	35	62	3	0
Cut down or stop smoking	64	33	3	0
Cut down or stop drinking tuba or other alcoholic beverages	15	45	30	10

When asked if tuba contains alcohol, most participants (75%) responded “No.” Nearly half (48%) answered “True” to the statement: “Tuba or other alcoholic beverages are good for you and the baby while you are pregnant.” Women who indicated that tuba or other alcoholic beverages have health benefits (48%) were asked to describe said benefits in open-ended format (Table 3). Similarly, participants who responded “False” (52%) selected from a variety of reasons supporting their belief and/or listed other reasons not captured by the multiple choice format (Table 3).

**Table 3:** Self-reported effects of drinking tuba or other alcoholic beverages during pregnancy.

What are some good effects of drinking tuba or other alcoholic beverages while pregnant?	*N*	%
Open-ended responses		
It increases red blood cell count	23	23
It increases lactation	14	14
It keeps the baby healthy	8	8
It provides good nutrition	7	7
It keeps the mother healthy	5	5
It improves the labor process	5	5
It has anti-parasitic properties	2	2
It helps the mother sleep	1	1
It relaxes the mother	1	1
What are some bad effects of drinking tuba or other alcoholic beverages while pregnant?	*N*	%
Closed-ended responses		
The baby may not grow as well and will be born smaller	39	39
Miscarriage	12	12
The baby can have cognitive or “thinking” problems	10	10
Stillbirth	9	9
The baby can have behavioral problems	7	7
Open-ended responses		
It causes dizziness/nausea	3	3
The child may become dependent on tuba	1	1
The mother has an aversion to the smell of tuba	1	1
It makes the child sick more easily	1	1
It causes hyperacidity	1	1
It causes congenital anomalies	1	1
It interferes with anesthesia	1	1
It does not affect the baby	1	1

**Figure 2:**
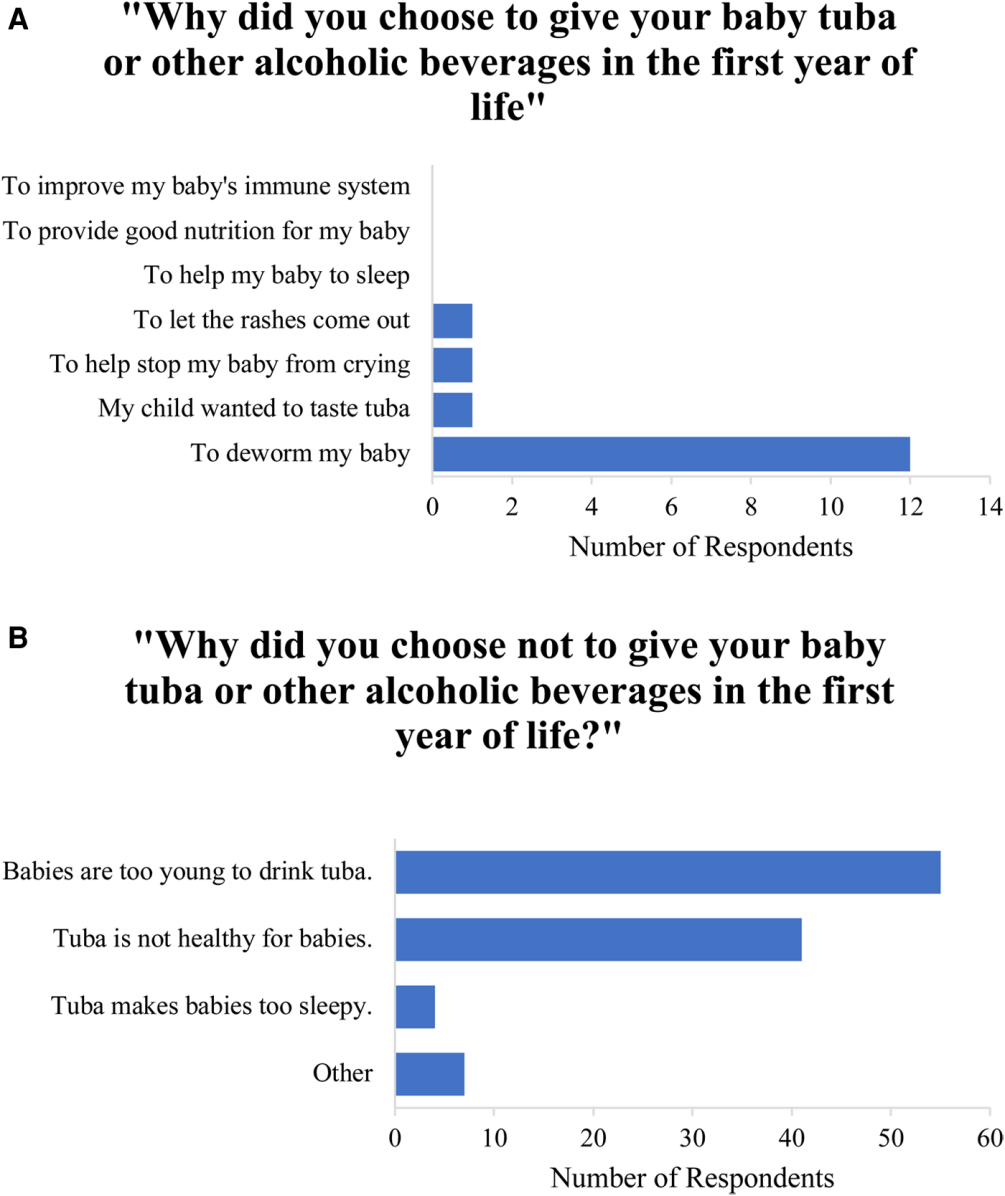
(**A**) Participants’ cited reasons for feeding tuba or other alcohol to their babies. (**B**) Participants’ cited reasons for not feeding tuba or other alcohol to their babies.

Overall, 15% (*N* = 15) of participants reported giving tuba or another alcoholic beverage to their youngest child within the first year of life. Of these respondents, 100% reported giving their child tuba and no other alcoholic beverage. Of 15 participants who gave their child tuba, most (87%) gave their child less than 1 teaspoon of tuba per day. None reported giving their child more than 2 teaspoons of tuba per day. The reasons participants gave regarding whether or not to feed their baby tuba are described in Figures 2A,B.

**Figure 3:**
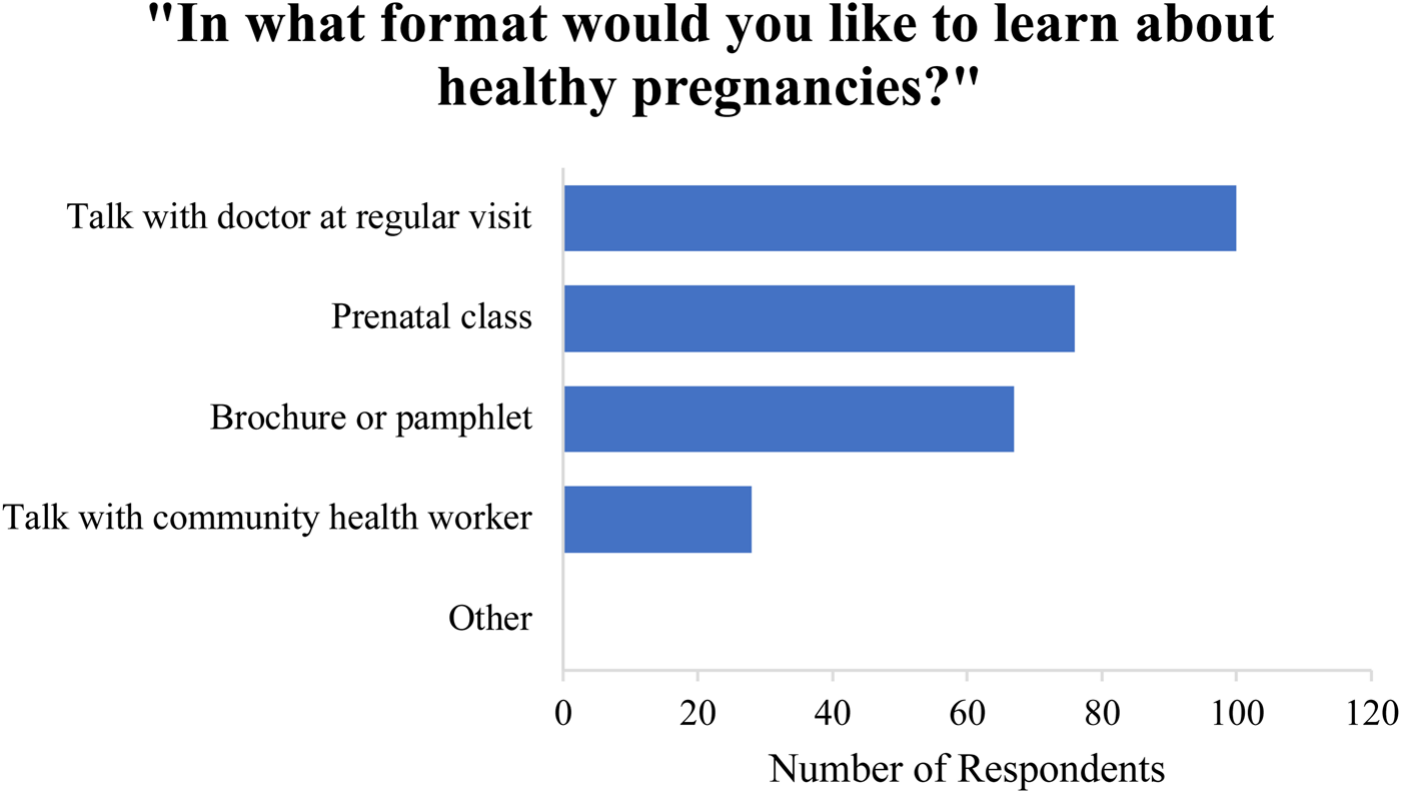
Preferred modalities by which participants would like to learn more about healthy pregnancies.

Participants were asked if their families or friends encouraged them to drink tuba while pregnant and most (59%) responded “yes.” In response to “Did your doctor explain to you the effects of drinking tuba during pregnancy?”, 20.0% of participants (*n* = 18) reported “yes” while 48.9% (*n* = 44) reported “no.” Overall, 31.1% of participants (*n* = 28) reported never having visited a doctor during the course of their pregnancy**.** Nearly all mothers (98%) reported “yes” when asked if they would cut back on their drinking if they were told that tuba or other alcoholic beverages have negative effects on them and their unborn child. Two participants (2%) answered “no”, stating that they never personally experienced any side effects from prenatal tuba consumption. However, all of the women surveyed stated they would like to learn more about how to keep their baby/pregnancy healthy, with preferred modalities presented in Figure 3.

**Figure 4:**
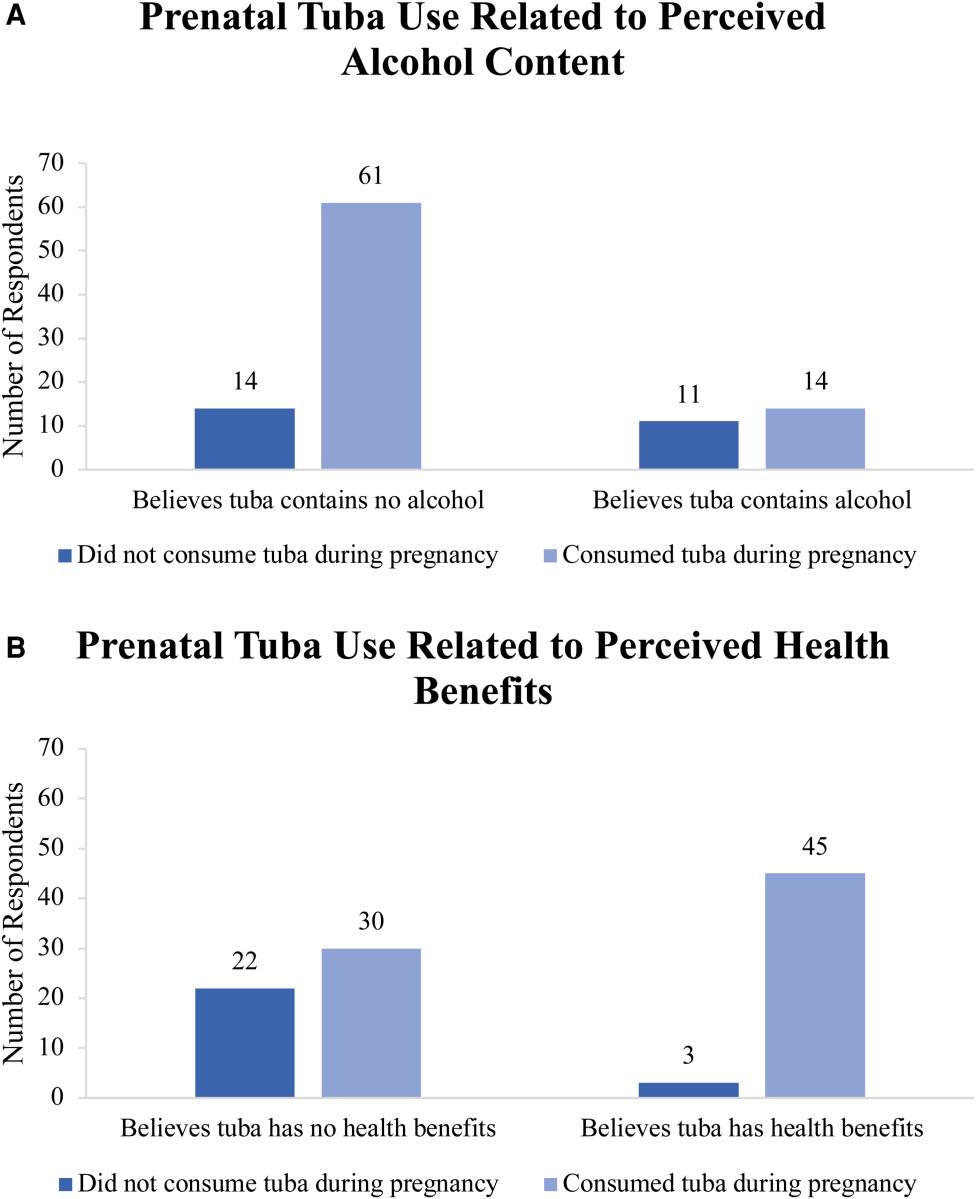
(**A**) Prenatal tuba use among participants as related to their perception of alcohol content in tuba. (**B**) Prenatal tuba use among participants as related to their perceived health benefits of tuba.

Women who believed tuba contains no alcohol were more likely to drink tuba (81.3%) than women who believed tuba contains alcohol (56.0%), *X*^2^ (1, *N* = 100) = 6.41, *p *= .011 (Figure 4A). Women who drank tuba during pregnancy were more likely to believe tuba has health benefits (60%) than women who did not drink tuba during pregnancy (12%), Fisher's exact *p* < .05 (Figure 4B).

## Discussion

Though there remains some controversy regarding the risks to the fetus with consumption of low levels of alcohol pre-natally (23), there is currently no known safe threshold for prenatal alcohol consumption (24, 25). As such, it is particularly concerning that 75% of women in this cohort reported drinking alcohol while they were pregnant. These findings are consistent with the self-reported rates of prenatal alcohol consumption from a previous study conducted in Leyte with a different group of pregnant women, bolstering the reliability of these responses (20). Overall, we found that a high proportion of women continued to drink during pregnancy and that many were not aware of the harmful effects of alcohol use during pregnancy.

When asked about the importance of various health-related activities during pregnancy, a contrast was noted between activities related to alcohol consumption and all other health-related activities. Greater than 95% of mothers considered other behavioral changes to be either “important” or “very important.” In contrast, only 60% of participants considered decreasing the consumption of tuba or other alcoholic beverages during pregnancy “important” or “very important.” This suggests that tuba or other alcoholic beverages are not viewed in the same light as other unhealthy practices (e.g., smoking) and may not be prioritized for behavioral change. Given many women may not be aware that tuba contains alcohol, some may have also de-emphasized the importance of reducing consumption. Other studies in LMICs have also found that women are not aware of potential harms of alcohol during pregnancy and may, therefore, not modify these behaviors (12, 14, 16).

The majority of women who endorsed drinking “tuba or other alcoholic beverages” in the study exclusively drank tuba and no other alcoholic beverage. The majority of mothers did not believe tuba contained alcohol, and there was a significant association between self-reported prenatal tuba consumption and the belief that tuba does not contain alcohol. Of the mothers who believed that tuba or other alcoholic beverages were good for them and their baby, most (78%) deemed it “healthy” to consume three or more glasses of tuba/alcohol per week. This suggests a lack of understanding of the negative effects of tuba and/or other alcoholic beverages on the developing fetus, likely leading to a lack of moderation of prenatal alcohol intake. As well, these findings suggest that misinformation regarding the alcohol content of tuba may play a key role in the consumption of alcohol during pregnancy in Leyte. Unregulated brews such as tuba are not easily monitored, and consumers cannot rely on such brews having standardized pregnancy warning labels, such as those seen on government-regulated alcoholic beverages, which is an issue globally (19). Broader education campaigns and targeted screening provided during prenatal care visits could serve as platforms to disseminate information on the dangers of prenatal tuba consumption.

Recent systematic reviews also suggest multiple reasons for continued pre-natal alcohol use that likely vary across settings but with similar themes emerging. These include lack of awareness of harm and even perceived benefit, medical advice to continue, peer and cultural influences, and others (14). Our findings are concordant with many of these reasons. More than half of respondents reported being encouraged by family or friends to drink tuba while they were pregnant, suggesting that external influences may play a role in prenatal alcohol consumption in the community. Moreover, nearly half of the study participants reported that their doctor never explained the effects of drinking tuba during pregnancy. Several different possibilities could explain this finding: (1) tuba consumption during pregnancy is an under-recognized practice, (2) healthcare providers are also unaware of tuba's alcoholic content and its subsequent harm to the developing fetus, (3) there exists no standardized prenatal care practice in Leyte for healthcare providers to screen for tuba or other alcohol use, or (4) the majority of women in the Philippines receive prenatal care from midwives, and participants only answered the question as it related to seeing a “doctor (26). Further work surveying healthcare professionals in Leyte would help clarify which of these scenarios is most likely.

In studies in other LMICs, women often cite health benefits of alcohol to promote lactation (14) and directly benefit young children when given to them. In our study, alcohol exposure extended beyond pregnancy with 15% of mothers giving their children tuba during their first year of life. Deworming was the reason most often cited for this practice. The frequency of this response suggests an underlying cultural belief that tuba contains antiparasitic properties, despite there being no evidence that alcohol exhibits antiparasitic effects *in vivo.* This belief may be tied to the significant burden of schistosomiasis, a disease caused by parasitic trematode worms, in the region (27).

All of the women surveyed (100%) stated that they would like to learn more about how to keep their pregnancy and baby healthy. Additionally, nearly all mothers (98%) reported that they would reduce their tuba or alcohol consumption if they were told that tuba or alcohol has been shown to have negative effects on them and their unborn child. Such high response rates highlight mothers' underlying desire to maintain healthy pregnancies as well as their willingness to modify their behavior with appropriate educational intervention. It also supports a key tenet of behavioral change, specifically motivation to change. When asked which modalities they would most like to learn from with regards to maternal-fetal health, all participants selected “talk with doctor during regular appointment,” emphasizing one potential avenue of public health intervention. In this setting, other healthcare providers such as midwives would also need to be engaged as they provide a large proportion of prenatal care in Leyte.

There are currently few studies of KAPB regarding prenatal alcohol exposure in low- and middle-income countries (LMICs), such as The Philippines. The findings from this study may not be solely limited to the communities of Leyte; they likely apply more broadly to resource-poor settings throughout the world with cultures that have similar alcoholic beverages consumed prenatally. It has been well-documented that numerous cultures consume similar customary wines, notably during pregnancy. In India, an alcoholic palm wine known as toddy is consumed during pregnancy and has been shown in rat fetuses and pregnant rats to cause hyperlipidemia, hypoglycemia, and alcohol-related liver toxicity at rates above that of only ethanol consumption (28, 29). In the Bendel State of Nigeria, pregnant women consume a palm wine that is believed to increase lactation despite the general lack of knowledge of its alcohol content (30). These examples illustrate the need for a deeper understanding of the KAPB of pregnant women who consume various forms of alcohol, whether or not they are aware of the alcoholic nature of the beverages they drink. It also suggests that in resource-poor settings, the prevalence of largely unregulated “home brews” makes it more difficult to track consumption and provide formal warnings on labels. The study further supports the need for more focused public health and educational interventions related to prenatal alcohol consumption specific to resource-limited settings to ultimately to reduce FASD morbidity globally.

This study has some limitations. While a pilot study was performed to optimize comprehension and reliability, we did not conduct extensive test-retest reliability assessments. Although we adapted an already existing, validated survey in an attempt to improve validity, the initial instrument required extensive modification in order to meet literacy levels and to adequately capture the knowledge, attitudes, practices, and beliefs specific to our target population. Further, since this KAPB survey was conducted postnatally, we could not validate reported prenatal practices against a gold standard measure of current alcohol consumption such as PEth (phosphatidylethanol) testing (31). In addition, participants were surveyed anywhere from 6 months to 24 months postpartum, which may have impacted accuracy of responses regarding prenatal alcohol consumption practices, with women asked later perhaps having lower recollection of practices. Finally, response bias is always a possible concern when dealing with behaviors surrounding alcohol use, though such biases are more likely when there is potential stigma associated with a specific response (32). Such biases can lead to inaccurate estimations of alcohol consumption during pregnancy. This is less likely in the current study as prenatal tuba consumption does not appear to be highly stigmatized in Leyte based on both the high rates of reported use and the misconception that tuba does not contain alcohol.

The finding that most women believed tuba did not contain alcohol hindered interpretation of several questions which asked about “tuba or other alcoholic beverages.” Participants may have found it difficult to comprehend questions which grouped tuba into the same category as alcoholic beverages. For example, nearly half (48%) of participants answered “True” to the statement: “Tuba or other alcoholic beverages are good for you and the baby while you are pregnant.” Given that many participants did not regard tuba as an alcoholic beverage, it is difficult to assess whether participants were addressing tuba, alcoholic beverages, or both items in their answers. It should also be noted, however, that we split many analyses based on whether women believed tuba contained alcohol or not and it was still the case that 56% of women who believed tuba contained alcohol continued to drink tuba during pregnancy. In future studies, these should be separated.

## Conclusion

Misinformation about tuba as an alcoholic beverage appears to play a role in the practices surrounding maternal and pediatric tuba consumption in Leyte, The Philippines. Most participants in our study reported consumption of alcohol during pregnancy. Of note, the majority of these mothers consumed exclusively tuba during their pregnancy and most of them did not acknowledge tuba to be an alcoholic beverage. This suggests key areas for education, especially since women stated they would change behaviors if doing so would improve the health of their babies. Given tuba's 7%–8% alcohol content and that there is no known safe threshold for alcohol consumption during pregnancy, the high rate of tuba consumption in our study highlights a serious risk of FASD in the offspring of the population surveyed and likely many populations globally that consume home brews. Future work should focus on incorporating tuba screening into already existing structures for alcohol and tobacco smoking screening at prenatal visits. Data from this study can inform local health departments in the creation of health education materials and/or programs addressing prenatal alcohol use for women of childbearing age.

## Data Availability

The raw data supporting the conclusions of this article will be made available by the authors, without undue reservation.
