# Supplementary material for: Knowledge, attitudes, practices, and beliefs regarding prenatal alcohol consumption among women in Leyte, the Philippines
Source: Front Glob Womens Health. 2023 Aug 9;4:1156681. doi: 10.3389/fgwh.2023.1156681 (PMC10445536; doi:10.3389/fgwh.2023.1156681)
Supplement: Supplementary file 1 [file Datasheet1.docx]

**Knowledge, Attitudes, Practices, and Beliefs Regarding Prenatal Alcohol Consumption Among Women in Leyte, The Philippines**

**APPENDIX**

Prenatal Alcohol Use KAPB Survey in Waray and English

1. Paunanho ka importante ha imo nga magiging maupay it imo kahimtang dida han imo pagburod nga makaka-anak ka hin malibsog nga bata?

How important was it for you to be healthy during your pregnancy to make it more likely for your baby to be born healthy?

- 1. Duro gud nga kinahanglan / Very important
  2. Kinahanglan / Important
  3. Dire gud duro kinahanglan / Not very important
  4. Dire man kinahanglan / Not important at all

Mayda ko babasahon parte han mga burod an mga pama-agi nga makakabulig ngan bangin dire makabulig para maka-anak hin mahimsog nga bata. Sumati ako kun ha imo pag-abat in duro gud nga kinahanglan, kinahanglan, dire gud duro kinahanglan ngan dire gud kinahanglan.

I’m going to read some things that pregnant women might or might not do to make it more likely that their baby is born healthy. Tell me if you feel that these things are very important, important, not very important, or not important at all.

1. Pagbisita ha usa ka doktor

Visit a doctor or health professional

- 1. Duro gud nga kinahanglan / Very important
  2. Kinahanglan / Important
  3. Dire gud duro kinahanglan / Not very important
  4. Dire man kinahanglan / Not important at all

1. Pagka-on hin masustansya nga karaunon

Eat well/have good nutrition

- 1. Duro gud nga kinahanglan / Very important
  2. Kinahanglan / Important
  3. Dire gud duro kinahanglan / Not very important
  4. Dire man kinahanglan / Not important at all

1. Pisikal nga burohaton

Exercise/Perform physical activity

- 1. Duro gud nga kinahanglan / Very important
  2. Kinahanglan / Important
  3. Dire gud duro kinahanglan / Not very important
  4. Dire man kinahanglan / Not important at all

1. Pag-iban o pag-undang hin pagsigarilyo

Cut down or stop smoking

- 1. Duro gud nga kinahanglan / Very important
  2. Kinahanglan / Important
  3. Dire gud duro kinahanglan / Not very important
  4. Dire man kinahanglan / Not important at all

1. Pag-iban o pag-undang hin pag-inom hin tuba o iba pa nga makahurubog nga irimnon

Cut down or stop drinking tuba or other alcoholic beverages

- 1. Duro gud nga kinahanglan / Very important
  2. Kinahanglan / Important
  3. Dire gud duro kinahanglan / Not very important
  4. Dire man kinahanglan / Not important at all

1. Mayda ba nakasalakot nga alkohol it tuba? Mayda o Waray

Does tuba contain alcohol? Yes or No

8.1 Dida han imo pagburod,naka-inom ka ba hin tuba o iba pa nga makahurubog nga irimnon?

Naka-inom o Waray

During your pregnancy, did you drink tuba or other alcoholic beverages? Yes or No

8.2 Kun naka-inom, ano nga klase nga makahurubog nga irimnon?

If Yes, what kind of alcohol did you drink?

- 1. Tuba la (Pira na ka-tuig) / Tuba alone (specify age of tuba):
  2. Tuba nga mayda cola (Pira ka-tuig an tuba) / Tuba with Cola (specify age of tuba):
  3. Beer / Beer
  4. Gin / Gin
  5. Wine / Wine
  6. Rum / Rum
  7. Iba pa: / Other (specify):

8.3 Kun naka-inom,pira man adto ka baso ha usa ka semana?

If Yes, how many glasses did you drink a week?

- 1. Usa ngada duha / 1-2
  2. Tulo ngada lima / 3-5
  3. Unom ngada walo / 6-8
  4. Siyam ngada napulo / 9-10
  5. Sobra napulo / More than 10

8.4 Kun naka-inom, pira ka na ka-tuig nag-iininom hin tuba o iba pa nga makahurubog nga

irimnon?

If Yes, how many years have you been drinking tuba or other alcoholic beverages?

1. Kulang hin usa ka-tuig / Less than a year
2. Usa ngada lima / 1-5
3. Unom ngada napulo / 6-10
4. Onse ngada kinse / 11-15
5. Dise sais ngada beinte / 16-20
   1. Sobra beinte / More than 20

8.5 Kun waray, naka-inom ka ba hin tuba o iba pa nga makahurubog nga irimnon ha naglabay

nga panahon? Naka-inom o Waray

If No, did you drink tuba or other alcoholic beverages in the past? Yes or No

8.6 Kun Oo it imo baton han una nga pakiana (8.5), pira ka baso hit usa ka semana hadin naglabay nga panahon?

If Yes to the previous question (8.5), how many glasses did you drink a week in the past?

1. Usa ngada duha / 1-2
2. Tulo ngada lima / 3-5
3. Unom ngada walo / 6-8
4. Siyam ngada napulo / 9-10
5. Sobra napulo / More than 10
   1. Tinuod o Dire: Maupay ba it tuba o iba pa nga makahurubog nga irimnon ha imo ngan ha

imo bata samtang burod ka?

True or false: Tuba or other alcoholic beverages are good for you and the baby while you are pregnant.

- 1. Kun tinuod, ano an naging maupay nga epekto ha imo pag-ininom hin tuba o iba pa nga

makahurubog nga irimnon samtang burod ka?

If True, what are some good effects of drinking tuba or other alcoholic beverages while pregnant?

- 1. Kun tinuod, pira man ka baso nga tuba o iba pa nga makahurubog nga irimnon ha usa ka

semana it makaka-upay panlawas ha usa ka burod?

If True, how many glasses a week of tuba or other alcoholic beverages is healthy for a pregnant woman to drink?

1. Usa ngada duha / 1-2
2. Tulo ngada lima / 3-5
3. Unom ngada walo / 6-8
4. Siyam ngada napulo / 9-10
5. Sobra napulo / More than 10
   1. Kun dire, anuman it maraot nga epekto it pag-ininom hin tuba o iba pa nga makahurubog

nga irimnon samtang burod ka? (Pili-a iton imo mga baton.)

If False, what are some bad effects of drinking tuba or other alcoholic beverages while pregnant? (Choose all that apply.)

- 1. Dire maupay it pagtubo ngan kulang hin timbang it imo anak

The baby may not grow as well and will be born smaller

- 1. Ma-aapektuhan an paminsar ngan panhuna-huna an imo anak

The baby can have cognitive or “thinking” problems

- 1. Magkakamay-ada hin problema pamatasan an imo anak

The baby can have behavioral problems

- 1. Nakuha-an (Namatayan hin bata ha sakob han tiyan kulang hin beinte ka semana)

Miscarriage (the baby dies while inside the womb for less than 20 weeks)

- 1. Nakuha-an (Namatayan hin bata ha sakob han tiyan lampas hin beinte ka semana)

Stillbirth (the baby dies while inside the womb for more than 20 weeks)

- 1. Iba pa nga epekto: / Other (specify):
  2. Ginpa-inom mo ba an imo anak hin tuba o iba pa nga makahurubog nga irimnon ha sakob

hin usa ka tuig han iya pagkatawo? Naka-inom o Waray

Did you give your baby who is in the study tuba or other alcoholic beverages during his/her

first year of life? Yes or No

10.2 Kun ginpa-inom, anuman an ginhatag?

If Yes, what did you give your baby?

1. Tuba la (Pira na ka-tuig): / Tuba alone (specify age of tuba):
2. Tuba nga mayda cola (Pira ka-tuig an tuba): / Tuba with Coca Cola (specify age of tuba):
3. Beer / Beer
4. Gin / Gin
5. Wine / Wine
6. Rum / Rum
7. Iba pa nga irimnon: / Other (specify):

10.3 Kun ginpa-inom, pira man adto kadamo?

If Yes, how much did you give your baby?

1. Kulang hin 1 ka kutsarita / Less than 1 teaspoon per day
2. Usa ngada duha ka kutsarita / 1-2 teaspoons per day
3. Tulo ngada ka lima ka kutsarita / 3-5 teaspoons per day
4. Sobra hin lima ka kutsarita / More than 5 teaspoons per day

10.4 Kun ginpa-inom, kay ano nimo gintagan hin tuba o iba pa nga makahurubog nga irimnon?

If Yes, why did you choose to give your baby tuba or other alcoholic beverages?

1. Para mangaturog / To help my baby to sleep
2. Para umundang pagtinuok / To help stop my baby from crying
3. Magmaupay an kahimtang panlawas han akon anak / To provide good nutrition for my baby
4. Pagpakusog han resistensiya han akon anak / To improve my baby’s immune system
5. Pagpurga han akon anak / To deworm my baby
6. Iba pa: / Other (specify):

10.5 Kun waray pa-inoma, kay anu nimo waray tagi hin tuba o iba pa nga makahurubog nga

irimnon? (Pili-a iton imo mga baton.)

If No, why did you choose not to give your baby tuba or other alcoholic beverages?

(Choose all that apply.)

1. Dire maupay it tuba ha bata. / Tuba is not healthy for babies.
2. Mangangaturog it akon anak. / Tuba makes babies too sleepy.
3. Dire pa puyde para ha iya edad. / Babies are too young to drink tuba.
4. Iba pa: / Other (specify):

11.1 Kun susumatan ko ikaw nga it tuba o iba pa nga makahurubog nga irimnon in mayda

maraot nga epekto ha imo ngan ha bata nga imo ginbuburod,maundang ka ba pag-ininom?

If I told you that tuba or other alcoholic beverages has been shown to have negative effects

on you and your unborn child, would you cut back on your drinking?

- 1. Oo / Yes
  2. Dire / No

11.2 Kun dire, alayon pag-eskplikar kon kay ano diri nimo na-uundang it imo pag-inom?

If No, could you explain why you would not cut back on your drinking?

1. Gin-pupursige ka ba pagpa-inom hin tuba han burod ka pa? Oo o Dire

Did any of your loved ones (family or friends) encourage you to drink tuba while you were pregnant? Yes or No

1. An imo doktor nag-eksplikar ha imo iton epekto hit pag-inom hin tuba kon burod ka?

Did your doctor explain to you the effects of drinking tuba during pregnancy?

- 1. Oo / Yes
  2. Dire / No
  3. Waray makadto hin doktor han burod ako. / Did not visit doctor during pregnancy.

14.1 Naruyag ka ba mahibaro kun pa-unanhon maging mahimsog an imo anak ngan an imo

pagburod? Oo o Dire

Would you like to learn more about how to keep your baby/pregnancy healthy? Yes or No

14.2 Kun naruyag, pa-unanhon man nga mga pamaagi para mabaro hini?

If Yes, what format would you like to learn about this?

1. Magpakonsulta ha usa ka doctor / Talk with doctor during regular appointment
2. Pakig-istorya hin usa ka komadrona / Talk with community health worker during home visits
3. Pag-atinder hin klase para hit mga burod ngan nanay nga mayda gudti nga anak / Class with other pregnant women or mothers of young babies
4. Mga barasahon parte panlawas / Brochure or pamphlet
5. Iba pa: / Other (specify):
